# Surgery for recurrent biliary carcinoma: results for 27 recurrent cases

**DOI:** 10.1186/s12957-015-0507-8

**Published:** 2015-02-27

**Authors:** Takehiro Noji, Takahiro Tsuchikawa, Tomoko Mizota, Keisuke Okamura, Toru Nakamura, Eiji Tamoto, Toshiaki Shichinohe, Satoshi Hirano

**Affiliations:** Department of Gastroenterological Surgery II, Hokkaido University Graduate School of Medicine, Kita 15 Nishi 7, Kita-ku, Sapporo, Hokkaido 0608638 Japan

**Keywords:** Recurrent biliary malignancy, Extrahepatic cholangiocarcinoma, Gallbladder carcinoma, Surgery, Chemotherapy, Survival

## Abstract

**Background:**

Various chemotherapies have been used as best practice to treat recurrent biliary malignancies. Conversely, relatively few surgeries have been described for recurrent extrahepatic biliary carcinoma (RExBC), so whether surgery for RExBC is feasible has remained unclear. This retrospective study was conducted to evaluate the feasibility of surgery for RExBC.

**Methods:**

From February 2000 to January 2014, a total of 27 patients, comprising 18 patients with extrahepatic cholangiocarcinoma and 9 patients with gallbladder carcinoma, met our criteria for radical resection of RExBC (resection group). Sites of recurrence consisted of liver metastases (ten patients), local/percutaneous transhepatic cholangio drainage (PTCD) fistula recurrence (eight patients), bile duct recurrence (six patients), and lymph node recurrence (one patient). To evaluate the survival impact of resection, we compared 123 RExBC patients (resection group) with patients who received palliative care (palliative group).

**Results:**

Morbidity and mortality rates in the resection group were 6.6% and 0%, respectively. Overall cumulative 5-year survival rates were 23.5% in the resection group and 0% in the palliative group. Median survival time was 21.6 months in the resection group and 9.5 months in the palliative group, showing a significant difference (*p* < 0.01). No significant differences in cumulative survival were seen between extrahepatic cholangiocarcinoma and gallbladder carcinoma in the resection group. In addition, no significant differences were seen between liver metastases, bile duct recurrence, and local/percutaneous transhepatic biliary drainage (PTBD) fistula recurrence in the resection group.

**Conclusions:**

Surgery appears feasible for RExBC and offers longer survival for selected patients.

## Background

Long-term survival rates for extrahepatic biliary carcinoma (extrahepatic cholangiocarcinoma and gallbladder carcinoma) are thought to be improving. Five-year cumulative survival rates have been reported as 22 to 40% for extrahepatic cholangiocarcinoma and 8 to 65% for advanced gallbladder carcinoma [1-7]. For now, no evidence supports the use of adjuvant chemotherapy [8].

For recurrent biliary malignancies, various chemotherapies have been used as best practice [9]. Conversely, few surgeries for recurrent extrahepatic biliary carcinoma (RExBC) have been reported, because secondary (or more) resection for RExBC is known as an extremely demanding procedure. Most patients with extrahepatic biliary carcinoma undergo complex anatomical resection and reconstruction with radical lymphadenectomy around the hepatoduodenal ligament (skeletonization) in the primary surgery. Few reports have described surgical interventions for RExBC, and those have mostly been case reports [10-14]. Whether surgery for RExBC is feasible thus remains unclear.

We have performed aggressive surgical intervention for RExBC since 2000. This retrospective study was conducted to evaluate the feasibility of surgery for RExBC following radical operation.

## Methods

### Surgical indications for RExBC

Surgical indications for RExBC in our department at the time of the study were as follows:

A). All surgical intervention should be intended as R0 curative resection. Resectability was determined from several imaging modalities (computed tomography, magnetic resonance imaging, ultrasonography, endoscopic ultrasonography, ^18^ F-fluorodeoxy glucose positron emission tomography). If multiple peritoneal disseminations or multiple metastases that were unable to be resected with curative intent were found, palliative procedures (gastrointestinal bypass or exploratory laparotomy only) were selected.
B). All candidates for surgical intervention were observed for at least 3 months with or without chemotherapy before resection. In addition, all candidates for surgical intervention should have isolated metastatic lesions. If a new metastatic lesion was found during this period, surgical intervention was not selected.
C). All candidates should have performance status 0 or 1 according to Eastern Cooperative Oncology Group criteria [15]. We used our criteria for hepatectomy in biliary malignancies, as described previously [16]. Anatomical variation and tumour location were also considered in determining resectability.

### Patients

From February 2000 to January 2014, a total of 150 patients with RExBC were identified for whom follow-up of the complete clinical course in our department was available. Among these patients, a total of 27 patients (21 men, 6 women; median age 71 years; age range 45 to 83 years) met our surgical criteria for RExBC. The underlying pathology was extrahepatic cholangiocarcinoma in 18 patients and gallbladder carcinoma in 9 (resection group).

To evaluate the survival impact of resection for RExBC, we reviewed the remaining 123 unresectable patients (92 men, 31 women; median age 71 years; range 48 to 84 years) with RExBC (palliative group). This palliative group included eight patients who underwent exploratory laparotomy.

### Type of recurrence

Recurrent sites consisted of liver metastases, local/percutaneous transhepatic biliary drainage (PTBD) fistula recurrence, bile duct recurrence, lymph node recurrence, and lung metastasis. Participants comprised ten patients with liver metastases, eight patients with PTBD fistula recurrence, six patients with bile duct recurrence, one patient with lymph node recurrence, and two patients with lung metastasis in the resection group, respectively (Table 1).

**Table 1:** **Patient characteristics in the resection and palliative groups**

**Characteristics**	**Resection group (** ***n*** **= 27)**	**Palliative group (** ***n*** **= 123)**	***p***
Age (years)^a^	71 (45 to 83)^b^	71 (48 to 84)^b^	0.77
Male/female (cases)	21/6	92/21	0.81
Primary disease (cases)			
Extrahepatic cholangiocarcinoma	18	96	0.22
Gallbladder carcinoma	9	27	
Recurrent site (cases)			
Liver	10	34	-
Bile duct	6	5	
Lymph node	1	14	
Local/PTBD fistula	8	26	
Lung	2	0	
Combined	0	36	
Other	0	8	
Chemotherapy (cases)	12	52	1
Disease-free interval (months)	25.1 (10.3 to 112.6)^b^	13.0 (1.8 to 124.2)^b^	<0.01

^a^Age at recurrence. ^b^Values represent median (range) for each parameter. PTBD, percutaneous transhepatic biliary drainage.

Concerning patients in the palliative group who underwent exploratory laparotomy, six patients with local/PTBD recurrence showed peritoneal dissemination, and the remaining two patients with lymph node recurrence showed multiple lymph node metastases that were unable to be resected completely.

### Surgical procedure

All resections were planned to have sufficient surgical margins. For liver metastases, several types of hepatectomy were performed. We planned various types of surgeries for local/PTBD fistula recurrence with sufficient margins. For bile duct recurrence, various major hepatectomies or pancreaticoduodenectomies were performed. For lymph nodal metastases, metastatic lymph node resection with concomitant resection of surrounding organ was performed. For lung metastases, partial lung resections were performed.

### Morbidity and mortality

The Clavien-Dindo classification was used for defining morbidity and mortality [17]. Postoperative complications ≥ IIIa in the Clavien-Dindo classification were defined as morbidity.

### Evaluation of survival

To evaluate the survival impact of RExBC, we calculated the length from the day on which recurrence was identified in this study.

### Statistics

Statistical calculations were performed using Stat Flex software (Artech, Osaka, Japan) and the ‘Exact Test’ produced by Prof. S. Aoki (http://aoki2.si.gunma-u.ac.jp/exact/exact.html). The chi-square test, Fisher’s exact, and Mann–Whitney *U* tests were used as appropriate. Cumulative survival after surgery was calculated using the Kaplan-Meier method. The log-rank test was used to compare cumulative survival. Values of *p* < 0.05 were considered significant.

### Consent

This study was approved by the local institutional review board of Hokkaido University Hospital. And written informed consent was obtained from the patient for the publication of this report and any accompanying images.

## Results

Two patients in the resection group underwent repeated resections for metastases. One patient with liver metastases underwent three resections, and the other patient with lung metastases underwent two resections. We performed 30 surgeries for the resection group, and 8 exploratory laparotomies in the palliative group. The resectability rate for the entire series was thus 79%.

Disease-free intervals (DFIs) in the resection and palliative groups were 25.1 months (range 10.3 to 112.6 months) and 13.0 months (range 1.8 to 124.2 months), respectively. Significant differences in DFI were seen between the two groups (*p* < 0.01, Table 1).

We applied 30 various surgical procedures for the 27 cases in the resection group. Procedures and surgical results for the resection group are shown in Table 2. Morbidity and mortality rates were 6.6% and 0%, respectively.

**Table 2:** **Surgical procedure and results in the resection group: 30 surgeries for 27 resection group patients**

**Characteristics**	**Surgeries (** ***n*** **= 30)**
Operative procedure (cases)	
Major hepatectomy	7
Non-anatomical hepatectomy	11
Pancreaticoduodenectomy	2
Lung resection	3
Tumour resection	2
Chest wall resection	1
Chest wall resection + non-anatomical hepatectomy	2
Non-anatomical hepatectomy + IVC/jejunum/colon/diaphragm resection	1
Para-aortic lymphadenectomy + adrenalectomy	1
Morbidity (Clavien-Dindo ≥ IIIa) (cases)	2
Mortality (cases)	0

IVC, inferior venous cava.

**Figure 1:**
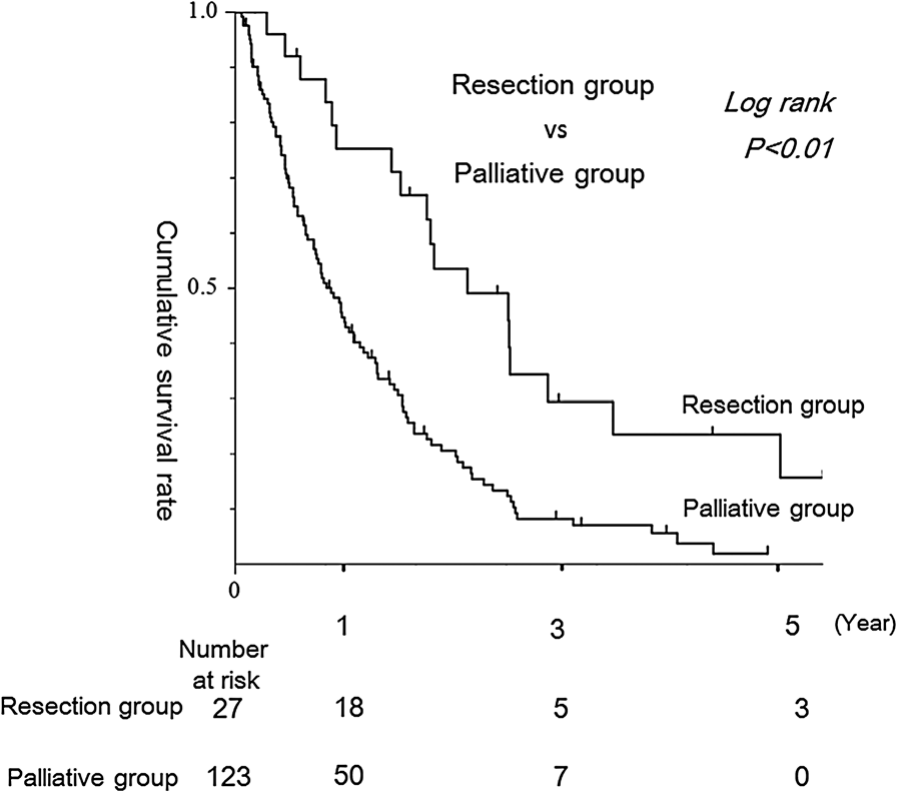
**Survival in the resection and palliative groups.** Cumulative 3- and 5-year survival rates in the patient resection group were 31% and 23%, respectively. Cumulative 3-year survival rate for patients in the palliative group was 0%. Significant differences were seen in survival between these two groups (*p* < 0.01).

The overall cumulative 5-year survival rate was 23.6% in the resection group and 0% in the palliative group. Median survival time was 21.6 months in the resection group and 9.5 months in the palliative group, showing a significant difference between groups (*p* < 0.01) (Figure 1).

Three patients in the resection group achieved 5-year survival (one patient with liver metastases, one with lymph node metastasis, and one with local/PTBD fistula recurrence).

**Figure 2:**
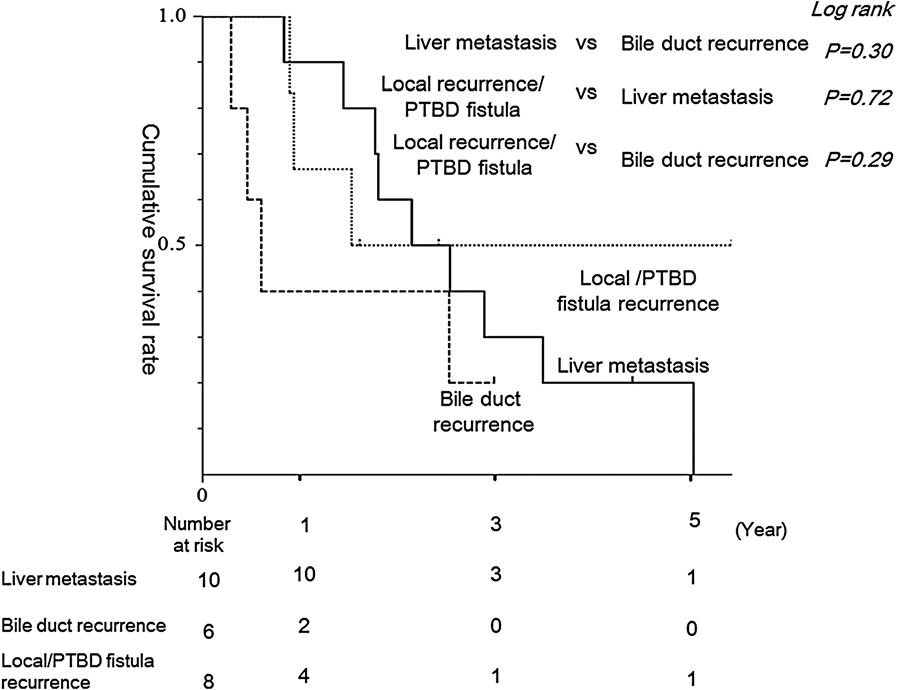
**Survival differences according to primary disease.** Cumulative 3- and 5-year survival rates for extrahepatic cholangiocarcinoma were 25% and 12%, respectively. Cumulative 3- and 5-year survival rates for gallbladder carcinoma were 43% each. No significant differences were seen in survival between these groups (*p* = 0.37).

No significant differences in cumulative survival were seen between extrahepatic cholangiocarcinoma and gallbladder carcinoma in the resection group. No significant differences between liver metastases, bile duct recurrence, or local/PTBD fistula recurrence were seen in the resection group (Figure 2). Likewise, no significant differences were seen in survival between DFI >24 and ≤24 months (*p* = 0.25).

**Figure 3:**
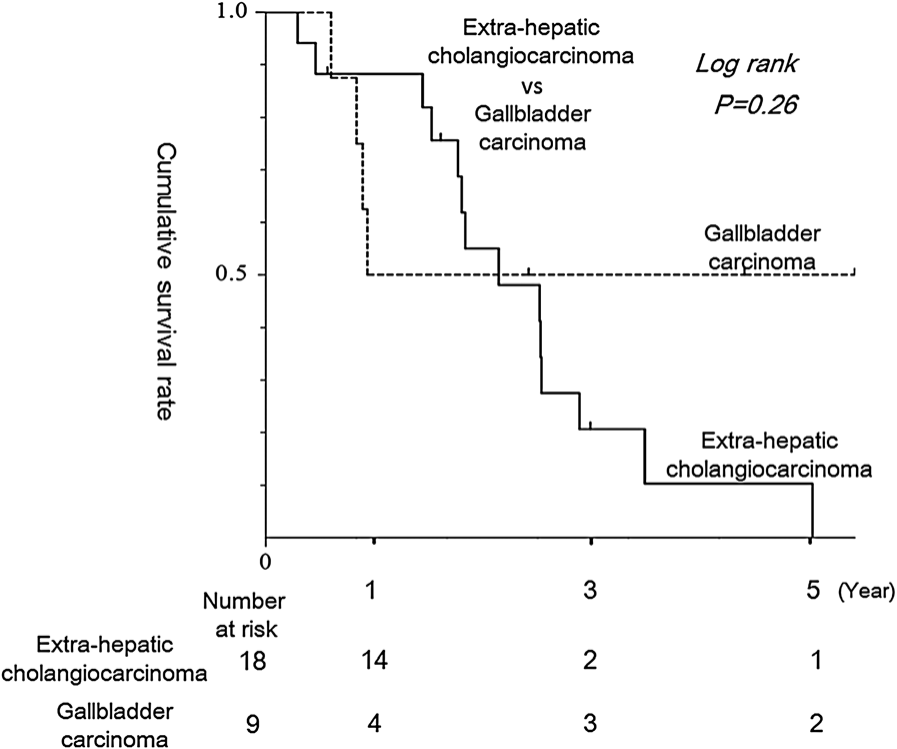
**Survival differences between local/PTBD fistula recurrence, liver metastasis, and bile duct recurrence.** Cumulative 3-year survival rates in local/PTBD fistula recurrence, liver metastasis, and bile duct recurrence were 50%, 33%, and 0%, respectively. Cumulative 3-year survival rate in local/PTBD fistula recurrence, liver metastasis, and bile duct recurrence were 50%, 17%, and 0%, respectively. No significant differences were seen in survival among these three groups.

No significant differences in survival were evident between extrahepatic cholangiocarcinoma and gallbladder carcinoma in the resection group patients (Figure 3).

## Discussion

In general, almost all patients with recurrent biliary malignancy are treated using various chemotherapies. Some trials have revealed that gemcitabine- or S-1-based chemotherapy could provide clinical benefit to patients with recurrent biliary malignancies [9,18-21]. However, these chemotherapies had few patients with long-term survival, and median survival times have been reported as less than 13 months [9,18-21].

One of the most important questions for RExBC is whether surgical intervention is feasible. Our results suggest that surgical intervention for RExBC might be worth considering. Cumulative 5-year survival rate in the resection group was 23.6%. We had low morbidity and no mortality in this series, and three patients survived for more than 5 years.

Concerning surgical indications for RExBC, only 1 report has referred to surgical results for RExBC; Song *et al.* reported surgical results for 27 patients with recurrent cholangiocarcinoma [22]. They showed that surgical resection for recurrent cholangiocarcinoma offers significant benefits for survival. However, because they merged recurrent intrahepatic cholangiocarcinoma (ICC) and recurrent extrahepatic cholangiocarcinoma and did not show operative results (operation time, operative bleeding, morbidity, or mortality) for their series, surgical indications for RExBC have remained unclear.

In terms of surgical intervention for recurrent ICC, few reports are available, and those reports have shown that surgical intervention for recurrent ICC would be feasible. Saiura *et al.* described five cases of repeat hepatectomy [23]. They had two patients with long-term survival. Okusaka *et al.* showed results for nine patients with various types of resection [19]. They performed curative resection for six of the nine patients and achieved relatively long survival for three. Fu *et al.* reported results for 19 patients with recurrent ICC treated using radiofrequency ablation (RFA) [24]. They showed a median overall survival of 30 months, and 1- and 3-year survival rates of 87.5% and 37.5%, respectively.

Quite substantial differences might be thought to exist between extrahepatic cholangiocarcinoma and gallbladder carcinoma. However, recent studies for recurrent biliary carcinoma, such as the ABC-01 study and BT22 study, have included various types of RExBC [19,21]. Furthermore, our data showed no significant differences between extrahepatic cholangiocarcinoma and gallbladder carcinoma in either the palliative or the resection group. We therefore considered that inclusion of both extrahepatic cholangiocarcinoma and gallbladder carcinoma in our study was proper.

For gastrointestinal malignancies, the efficacy of repeat hepatectomy for colorectal liver metastases is widely accepted; however, the benefits of such treatment for intrahepatic recurrence of gastric cancer liver metastasis remain unclear [25-27]. Most patients have been treated using various chemotherapies [28]. This situation is thought to be similar to that for RExBC. However, several reports have suggested that hepatectomy for metastatic lesions from gastric cancer would lead to long-term survival [25-27,29]. Takemura *et al.* showed results for 64 gastric cancer patients with liver metastasis. They showed that median overall survival was 34 months, with 3- and 5-year survival rates of 50 and 37%, respectively [26]. Baek *et al.* showed similar results [29]. Hwang *et al.* showed favourable median survival (27 months) for patients with RFA ± chemotherapy and no extrahepatic metastasis [25]. These results of recurrent ICC, gastric cancer, and our results for RExBC might suggest that surgical intervention for selected patients with RExBC is feasible.

The next question is that if surgical intervention for RExBC is indeed feasible, for whom and when should surgery be performed?

We used two major criteria for surgical intervention for RExBC. Nobody would doubt our criterion that all surgical procedures should be performed with the intention of R0 curative resection. In other words, every patient for whom R0 resection is considered possible would represent a surgical candidate.

Concerning the site of recurrence, we achieved successful resection for all candidates with bile duct recurrence and liver metastasis. Three of ten patients with liver metastasis were able to survive over 3 years after resection at the site of recurrence.

For bile duct recurrences, we have some doubts about surgical indications. We needed to perform demanding extended resection in the form of major hepatectomy or pancreaticoduodenectomy. However, the survival rate with bile duct recurrence tended to be lower than that with other types of recurrence, although no significant differences were identified (Figure 3).

We achieved lower resectability rates for patients with local/PTBD recurrence or lymph node recurrence than for patients with other recurrences. That means some patients were not adequately diagnosed in the preoperative survey. Our previous reports have shown that diagnosing lymph node metastasis preoperatively is difficult [30,31]. Diagnosing peritoneal disseminations preoperatively must likewise be difficult [32].

However, our data only included a small number of patients with peritoneal metastases or para-aortic lymph node metastasis for resection with curative intent. We achieved long survival for patients with para-aortic lymph node metastasis or local/PTBD fistula recurrence (‘localized’ peritoneal recurrence), and one patient achieved more than a 5-year survival. This suggests that surgical intervention is also feasible for selected patients with local/PTBD or lymph node recurrence.

When should surgery be performed? We observed all surgical candidates for at least 3 months with or without chemotherapy before surgery. This criterion was introduced based on our personal experience without objective evidence as such. However, we believe this criterion could allow exclusion of patients with a ‘rapidly growing tumor’ or patients with a ‘widely disseminating tumor.’

With regard to the timing of surgery, no significant criteria have been described in recurrent ICC series [19,23,24]. On the other hand, in patients with liver metastases from gastric cancer, Takemura identified disease-free interval, tumour size, and primary tumour stage as prognostic factors for survival [26,27]. Our data showed no significant effect of operative interval on survival.

Our criteria for RExBC led us to a 79% resectability rate, which was considered satisfactory.

This study had several limitations. First, it was a small, retrospective study and was not an intention-to-treat analysis. Whether surgical intervention is feasible thus remains in question. Second, whether neoadjuvant or adjuvant therapy is effective for resectable RExBC also remains unclear. Third, this study excluded carcinoma of the papilla of Vater. The feasibility of surgery for recurrent carcinoma of the papilla of Vater thus remains unclear.

These limitations would be best addressed in a future multi-centre study.

## Conclusions

In conclusion, despite these limitations, surgical intervention appears to offer improved survival in select patients with RExBC. Appropriate surgical indications and adjuvant therapy for RExBC should be investigated in a larger-scale study.

## Abbreviations

DFI: disease-free interval
ICC: intrahepatic cholangiocarcinoma
PTBD: percutaneous transhepatic biliary drainage
RFA: radiofrequency ablation
RExBC: recurrent extrahepatic biliary carcinoma
